# Bone-Preserving Management of Maxillary Cemento-Ossifying Fibroma With Orbital Floor Thinning: A Case Report

**DOI:** 10.7759/cureus.115351

**Published:** 2026-08-28

**Authors:** Francisco Javier Cano Palacios, Jael b Gomez Cabrales, Sergio Morales Acosta, Juan Pablo De la Fuente Martinez, Jose De Jesus Manfred Gomez Zazueta, Uriel A Guillen Morales

**Affiliations:** 1 General Surgery, Hospital Regional "Dr. Manuel Cárdenas de la Vega, Instituto de Seguridad y Servicios Sociales de los Trabajadores del Estado (ISSSTE), Culiacán, MEX; 2 Maxilofacial, Hospital General de Culiacán "Dr. Bernardo J. Gastélum", Culiacán, MEX; 3 General Surgery, Hospital Civil de Guadalajara "Fray Antonio Alcalde", Guadalajara, MEX; 4 General Surgery, High Specialty Regional Hospital of Veracruz, Veracruz, MEX; 5 General Surgery, Hospital General Regional (HGR) No. 1, Culiacán, MEX; 6 Plastic Surgery, Hospital General de Culiacán "Dr. Bernardo J. Gastélum", Culiacán, MEX

**Keywords:** cemento-ossifying fibroma, maxilar, shrunken orbit, tumor maxilar, weber-ferguson incision

## Abstract

Cemento-ossifying fibroma (COF) is a benign fibro-osseous neoplasm that most often affects the mandible and occurs predominantly in women during the third and fourth decades of life. Maxillary involvement is less common and may remain minimally symptomatic while expanding into the maxillary sinus. We present a 26-year-old woman with a six-month history of progressive right midface enlargement without pain, nasal obstruction, diplopia, visual symptoms, or dental mobility. Computed tomography demonstrated a well-circumscribed expansile right maxillary lesion measuring 44.9 × 42.5 × 31.9 mm, occupying a substantial portion of the maxillary sinus and extending toward a thinned but radiologically intact orbital floor. A preoperative incisional biopsy showed a fibrocellular spindle-cell stroma with cementum-like mineralized deposits, supporting the diagnosis of conventional COF. Through a Weber-Ferguson approach, the encapsulated lesion demonstrated a favorable cleavage plane and underwent macroscopically complete enucleation with peripheral osteotomy while preserving the orbital floor and residual maxillary bone. Immediate reconstruction was not required. At approximately three months of follow-up, wound healing remained satisfactory without infection, dehiscence, ectropion, alar retraction, visual symptoms, or other functional complications. Infraorbital hypoesthesia persisted as the only postoperative neurologic finding. Follow-up computed tomography was reviewed by the treating surgical team and demonstrated postoperative changes without a discrete residual or recurrent expansile lesion. This case highlights the importance of individualized intraoperative assessment of lesion boundaries and remaining structural support when considering a bone-preserving strategy. Although the early clinical and radiologic outcomes were favorable, the limited follow-up precludes conclusions regarding long-term recurrence, structural stability, or definitive sensory recovery.

## Introduction

Central cemento-ossifying fibroma (COF) is a benign, well-circumscribed fibro-osseous neoplasm characterized by the replacement of normal bone with a fibrous stroma containing variable amounts of bone- and cementum-like mineralized material. It predominantly affects women during the third and fourth decades of life and occurs more frequently in the mandible; maxillary and sinonasal involvement is less common [1].

Maxillary lesions may pose a diagnostic and therapeutic challenge due to their proximity to the maxillary sinus, nasal cavity, dentoalveolar structures, infraorbital nerve, and orbit. The available space within the maxillary sinus may permit relatively silent expansion, allowing the lesion to attain a considerable size before producing functional symptoms. Consequently, progressive facial asymmetry may be the first clinically relevant manifestation [2].

Current classification distinguishes conventional COF from juvenile trabecular ossifying fibroma and psammomatoid ossifying fibroma. These entities should not be used interchangeably because they differ in demographic distribution, microscopic mineralization patterns, biological behavior, and recurrence risk. Computed tomography commonly demonstrates a well-circumscribed expansile lesion, with internal density ranging from predominantly radiolucent to mixed or radiopaque depending on the degree of mineralization. Clinicoradiologic and histopathologic correlation is therefore essential to distinguish conventional COF from fibrous dysplasia and the trabecular and psammomatoid variants [3].

Surgical management should be individualized according to lesion boundaries, histologic subtype, anatomical extent, involvement of adjacent structures, and the amount of structurally supportive residual bone. Enucleation with peripheral osteotomy may be appropriate for selected well-circumscribed lesions, whereas wider resection may be necessary when macroscopically complete removal and anatomical preservation are not feasible [4]. We report a conventional maxillary COF with extensive maxillary sinus involvement and orbital floor thinning in which an encapsulated lesion with a favorable intraoperative cleavage plane permitted macroscopically complete enucleation, preservation of the orbital floor and residual maxillary bone, and avoidance of immediate reconstruction.

## Case presentation

**Figure 1:**
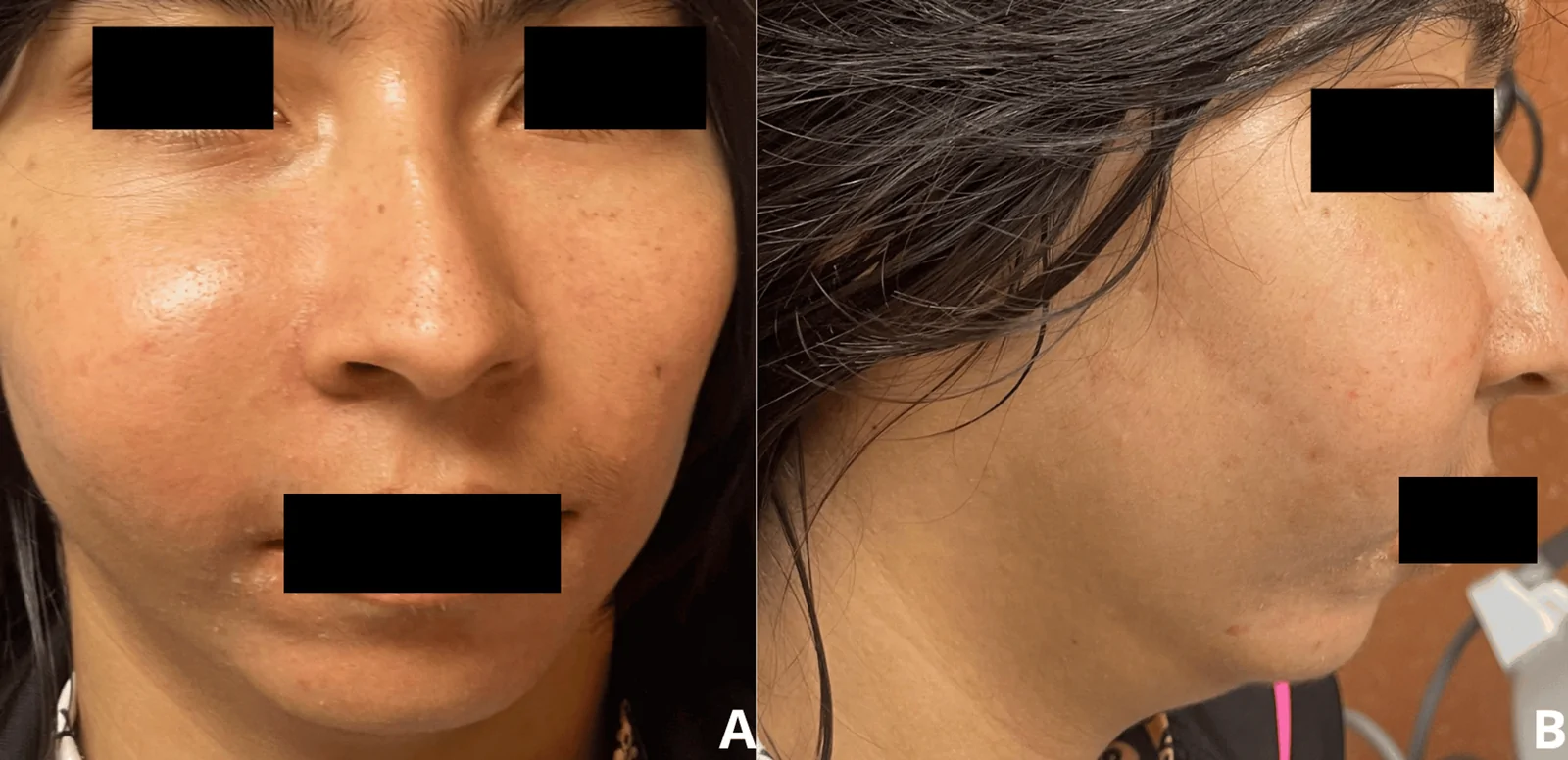
Preoperative facial appearance Unmarked preoperative photographs demonstrating right midface enlargement and facial asymmetry. (A) Frontal view. (B) Right lateral view demonstrating prominence of the right maxillary region.

A 26-year-old woman presented with progressive enlargement of the right midface over approximately six months. The growth was noticed primarily by her family. She denied pain, nasal obstruction, diplopia, visual symptoms, dental mobility, and other relevant functional limitations. Physical examination revealed right midface asymmetry centered over the maxillary region, as demonstrated in the unmarked preoperative frontal and right lateral photographs (Figure 1).

Intraoral examination showed intact maxillary mucosa without ulceration or clinically evident vestibular or palatal expansion. No dental mobility or clinically evident occlusal disturbance was identified. Formal pulp vitality testing was not performed or documented. No ocular or nasal involvement was clinically apparent. The initial clinical impression was a benign expansile maxillary lesion.

A preoperative incisional biopsy was performed. Gross examination showed an irregular, firm tissue fragment measuring 1.0 × 0.6 × 0.5 cm, with a solid, homogeneous, gray-white cut surface. Histopathologic examination revealed a benign fibro-osseous neoplasm composed of abundant spindle-shaped fibroblast-like cells, with a low mitotic index of two mitoses per 10 high-power fields and small rounded amphophilic cementum-like mineralized deposits. Non-neoplastic trabecular bone was identified at one edge of the biopsy. Following clinicoradiologic and histopathologic correlation, conventional COF was established as the working diagnosis before definitive surgery. No formal alternative preoperative diagnosis was documented.

**Figure 2:**
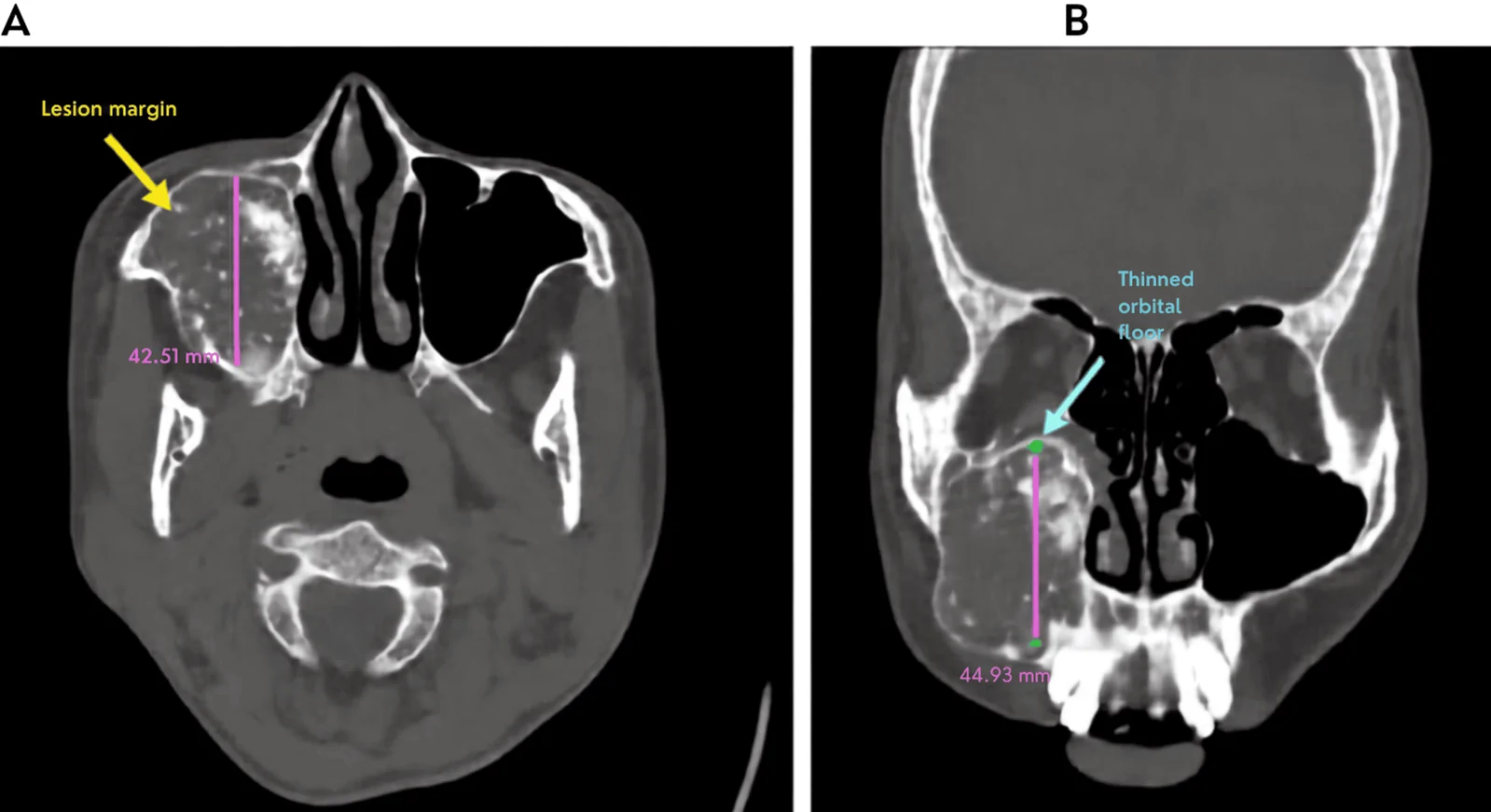
Preoperative computed tomography (A) Axial bone-window image showing the well-circumscribed margin of the mixed-density expansile lesion occupying the right maxillary sinus. (B) Coronal bone-window image demonstrating superior extension of the lesion and thinning of the right orbital floor, which remained radiologically intact.

Computed tomography demonstrated a well-circumscribed, mixed-density expansile lesion of the right maxilla measuring 44.9 × 42.5 × 31.9 mm, with internal mineralized components. The lesion occupied a substantial portion of the right maxillary sinus and produced expansion and remodeling of the surrounding maxillary bone. Superiorly, it extended toward the orbital floor, which was thinned but radiologically intact, with no evidence of perforation. Medially, the lesion approached the lateral wall of the nasal cavity without visible intranasal extension. Inferiorly, it was adjacent to the right maxillary dentoalveolar region; tooth-specific root displacement, resorption, and pulp vitality were not formally evaluated or documented (Figure 2).

**Figure 3:**
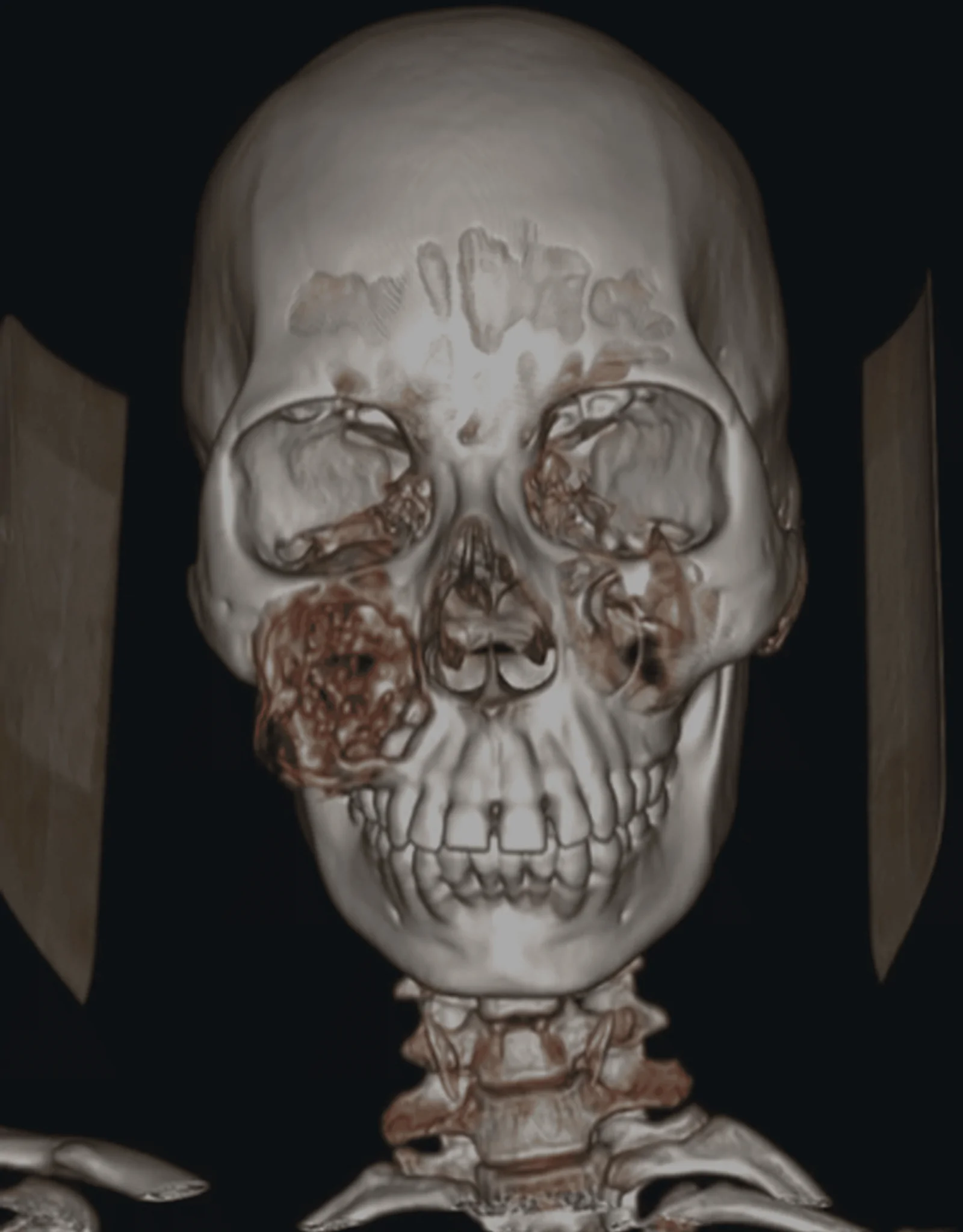
Three-dimensional reconstruction Three-dimensional reconstruction showing expansion and remodeling of the right maxilla caused by the expansile lesion.

Three-dimensional reconstruction further demonstrated expansion and remodeling of the right maxilla (Figure 3).

**Figure 4:**
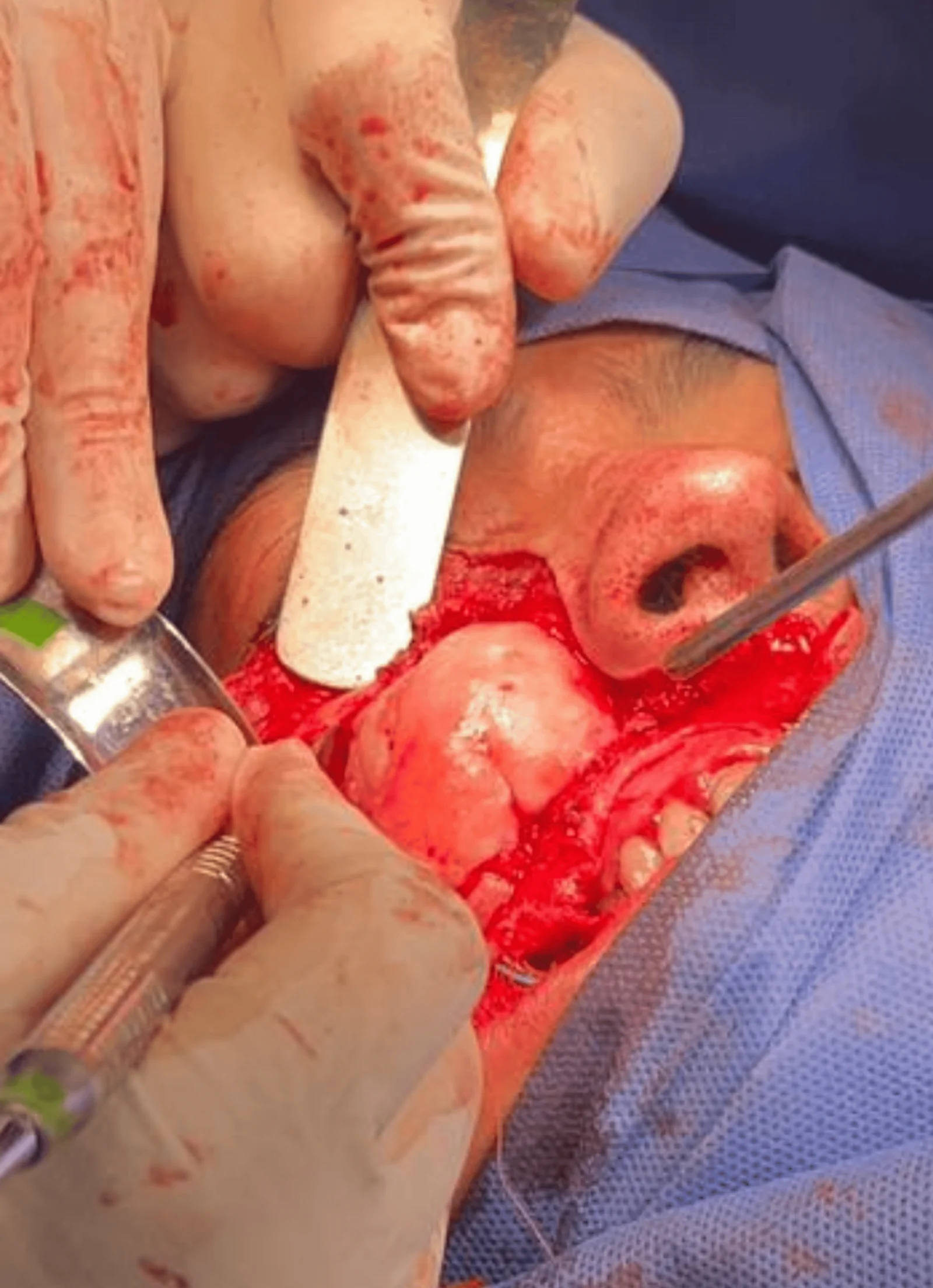
Intraoperative exposure of the maxillary lesion Intraoperative view through the Weber-Ferguson approach showing the exposed, well-circumscribed right maxillary lesion before enucleation. A macroscopically identifiable cleavage plane was observed between the lesion and the surrounding tissues.

Enucleation with peripheral osteotomy was selected instead of partial maxillectomy because the lesion was well circumscribed, the preoperative biopsy supported the diagnosis of conventional COF, the orbital floor remained intact, and preservation of sufficient native maxillary support appeared feasible. The patient underwent surgery on May 13, 2026. A Weber-Ferguson approach was selected to provide adequate exposure of the right maxilla, maxillary sinus, and infraorbital region. Intraoperatively, the lesion appeared well circumscribed and encapsulated, with a macroscopically identifiable cleavage plane separating it from the surrounding tissues (Figure 4). 

**Figure 5:**
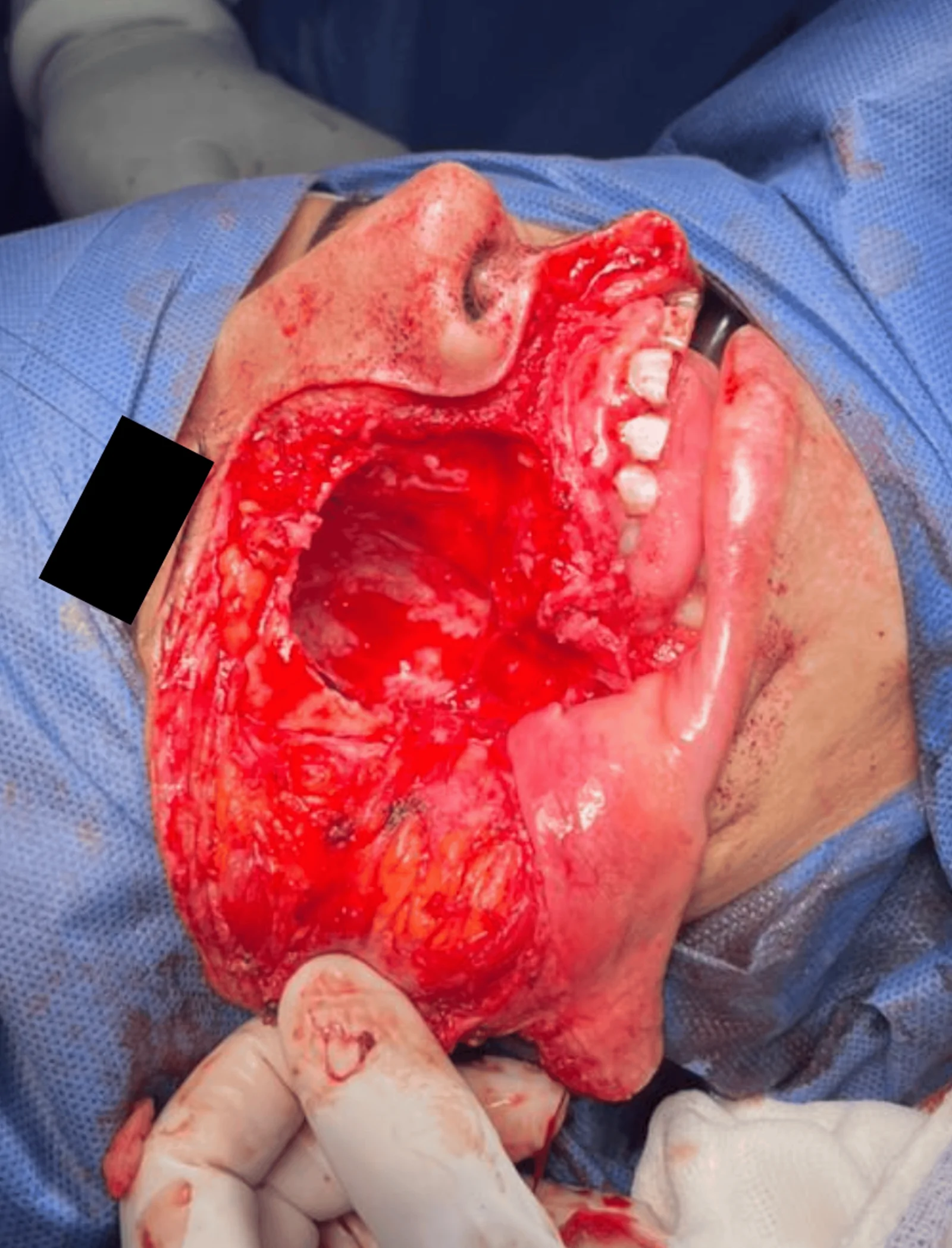
Intraoperative surgical bed after macroscopically complete enucleation Intraoperative view after macroscopically complete enucleation and peripheral osteotomy, showing the residual right maxillary bone, maxillary sinus cavity, and preserved orbital floor.

**Figure 6:**
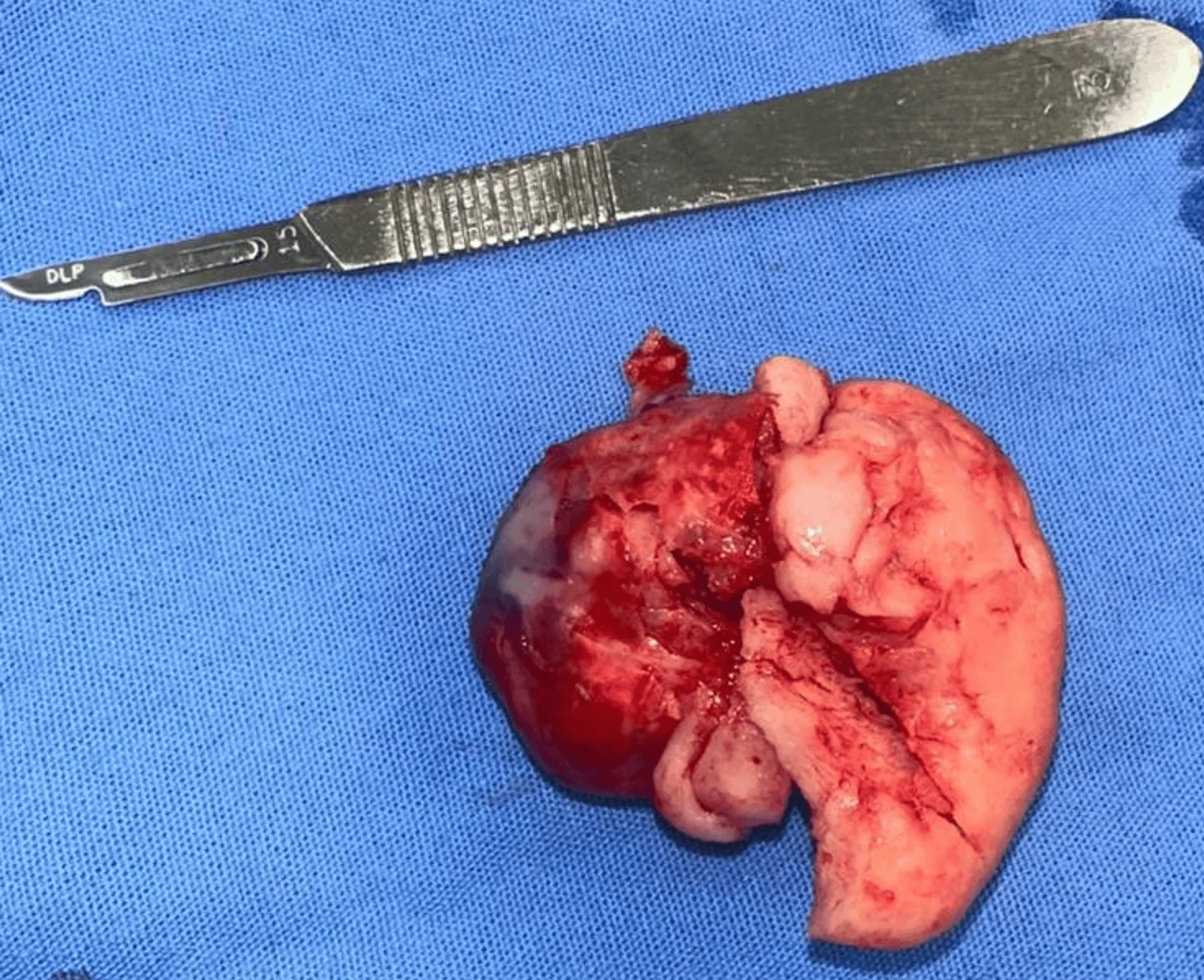
Fragmented surgical specimen Gross appearance of the fragmented surgical specimen obtained after macroscopically complete enucleation of the right maxillary lesion. Histologic assessment of the surgical margins was not possible because the specimen was received in multiple fragments.

Because the lesion could be separated macroscopically, the orbital floor remained structurally preserved, and sufficient residual maxillary bone was present, a bone-preserving approach was pursued instead of partial maxillectomy. Macroscopically complete enucleation with peripheral osteotomy was performed while preserving the orbital floor and the remaining maxillary bone (Figures 5-6).

Hemostasis was achieved, and an absorbable gelatin hemostatic sponge was placed in the surgical bed. No drains, fixation plates, bone grafts, local or free flaps, or immediate reconstructive procedures were required. Estimated blood loss was 400 mL, and no intraoperative complications were documented.

Definitive histopathologic examination of the fragmented surgical specimen was compatible with COF and showed no evidence of malignancy. Because the surgical specimen was received in multiple fragments, histologic assessment of the surgical margins was not possible. Representative histopathological micrographs could not be included because the incisional biopsy and surgical specimen were processed by the pathology department of the referral hospital, which provided written diagnostic reports but did not provide digital microscopic images to the treating team.

**Figure 7:**
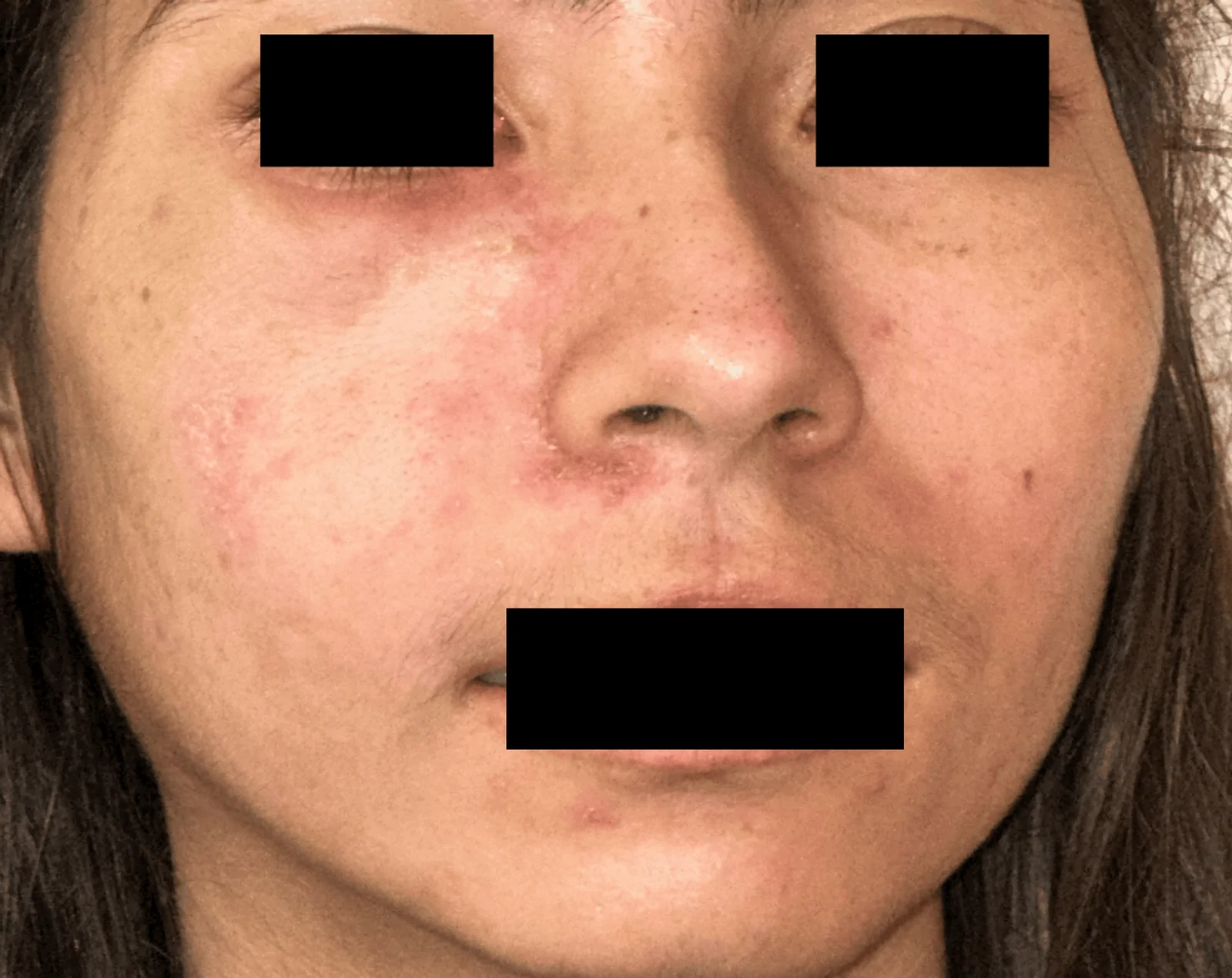
Three-month postoperative appearance Three-month postoperative clinical photograph documenting satisfactory wound healing without clinically evident dehiscence, ectropion, or alar retraction.

At approximately three months of follow-up, the patient maintained satisfactory wound healing without infection, dehiscence, ectropion, alar retraction, diplopia, visual symptoms, nasal obstruction, or other functional complications (Figure 7). The patient continued to report ipsilateral infraorbital hypoesthesia, which remained the only postoperative neurologic finding. No standardized or quantitative sensory examination was performed; therefore, the degree of sensory impairment and its change over time could not be objectively determined. Follow-up computed tomography was reviewed by the treating surgical team and demonstrated postoperative changes without a discrete residual or recurrent expansile lesion.​​​​​​​ Nevertheless, the limited follow-up period remains insufficient to evaluate long-term recurrence, structural stability, bone remodeling, or definitive sensory recovery.

## Discussion

Maxillary COF is less frequent than mandibular COF but may present a clinically relevant surgical challenge because of its relationship with the maxillary sinus, nasal cavity, dentoalveolar structures, and orbit. In the series by Kuta et al., central lesions of the maxillary sinus presented as relatively large, well-circumscribed expansile masses on computed tomography [2].

A subsequent series of maxillary and sinonasal ossifying fibromas identified the maxillary sinus as the most frequently involved site within this anatomical subgroup and showed that open surgical access was often required for extensive lesions [5]. Similarly, MacDonald-Jankowski reported that most maxillary ossifying fibromas in the reviewed literature involved the maxillary antrum, emphasizing their tendency to expand into adjacent sinonasal spaces [6]. Our patient remained free of pain and major functional symptoms despite maxillary sinus involvement and orbital floor thinning, consistent with expansion into an available anatomic space rather than an overtly destructive clinical course [2,7].

Current classification distinguishes conventional COF from juvenile trabecular ossifying fibroma and psammomatoid ossifying fibroma. These terms should not be used interchangeably because the entities differ in demographic distribution, microscopic mineralization pattern, biological behavior, and recurrence risk [3,8]. In this patient, the well-circumscribed and encapsulated lesion, together with a fibrocellular spindle-cell stroma, low mitotic activity, and rounded cementum-like mineralized deposits, supported conventional COF. Fibrous dysplasia was less consistent with the findings because it generally blends with adjacent bone rather than forming an encapsulated lesion with a favorable surgical cleavage plane. The pathology report did not describe the immature osteoid trabeculae characteristic of juvenile trabecular ossifying fibroma or the concentric psammomatoid bodies characteristic of psammomatoid ossifying fibroma. Definitive classification nevertheless requires clinicoradiologic and histopathologic correlation [3,8].

The choice between conservative removal and wider resection should be individualized according to margin definition, histologic subtype, lesion extent, involvement of critical structures, and the amount and quality of residual supportive bone. Gautier et al. found comparable recurrence rates after conservative and radical surgery for conventional COF, whereas juvenile trabecular and psammomatoid variants demonstrated higher recurrence and more frequently required aggressive management [9]. These findings support consideration of bone preservation in selected conventional lesions when macroscopically complete removal can be achieved without compromising essential structural support. Although virtual surgical planning was not used in this case, it may provide additional value in selected complex maxillofacial lesions by facilitating preoperative assessment of bone preservation and potential reconstructive requirements [10].

In the present case, enucleation with peripheral osteotomy was selected instead of partial maxillectomy because the lesion was well circumscribed and encapsulated, the preoperative biopsy supported conventional COF, the orbital floor was thinned but remained intact, and sufficient residual maxillary bone appeared preservable. Intraoperative assessment demonstrated a favorable cleavage plane and adequate remaining structural support, permitting macroscopically complete enucleation while preserving the orbital floor and native maxillary bone. This decision was based on both preoperative findings and direct intraoperative assessment and should not be interpreted as a universal recommendation for maxillary COF [4,9].

The Weber-Ferguson approach provided broad exposure of the right maxilla, maxillary sinus, infraorbital region, and orbital floor. Nnko et al. reported management of a large maxillary ossifying fibroma through a similar approach [11]. Therefore, the distinctive educational contribution of the present case is not the use of the Weber-Ferguson incision itself, but the intraoperative selection of a bone-preserving strategy for an encapsulated conventional COF with orbital floor thinning, allowing preservation of native support without immediate reconstruction.

Immediate reconstruction was not required during the early postoperative period in this patient. This observation does not establish that reconstruction can be safely deferred in all comparable lesions or that long-term structural stability has been demonstrated. The decision should depend on lesion boundaries, histologic subtype, orbital floor integrity, remaining support, and intraoperative findings.

Infraorbital hypoesthesia was the only postoperative neurologic finding. The infraorbital nerve required identification and dissection to obtain adequate exposure and permit lesion removal. The patient continued to report ipsilateral infraorbital hypoesthesia at the three-month follow-up; however, no standardized or quantitative sensory assessment was performed. Therefore, the severity of the sensory deficit and any change over time could not be objectively determined. The limited follow-up permits description only of early healing and sensory findings and is insufficient to determine long-term recurrence, bone remodeling, structural stability, definitive sensory recovery, or late orbital and sinonasal complications. Continued clinical and radiologic surveillance remains necessary [5,6,9].

## Conclusions

This case describes an encapsulated conventional maxillary COF with maxillary sinus involvement and orbital floor thinning that was managed by macroscopically complete enucleation with peripheral osteotomy through a Weber-Ferguson approach. Intraoperative confirmation of a favorable cleavage plane, preservation of the orbital floor, and adequate residual maxillary support permitted a bone-preserving procedure without immediate reconstruction in this patient. At three-month follow-up, wound healing remained satisfactory, and follow-up computed tomography was reviewed by the treating surgical team and demonstrated postoperative changes without a discrete residual or recurrent expansile lesion; ipsilateral infraorbital hypoesthesia persisted. These findings represent an individualized surgical decision and an early postoperative observation rather than evidence supporting a general treatment recommendation or long-term treatment success. Continued clinical and radiologic surveillance is required to evaluate recurrence, structural stability, bone remodeling, and sensory recovery.
